# Protective effects of wogonin on lipopolysaccharide-induced inflammation and apoptosis of lung epithelial cells and its possible mechanisms

**DOI:** 10.1186/s12938-021-00965-6

**Published:** 2021-12-14

**Authors:** Jinlin Ge, Huanhuan Yang, Yufeng Zeng, Yunjie Liu

**Affiliations:** 1 Department of Pulmonary and Critical Care Medicine, Wenzhou Hospital of Integrated Traditional Chinese and Western Medicine, Wenzhou, 325000 Zhejiang Province China; 2 Department of Respiratory and Critical Care Medicine, The Second People’s Hospital of Nantong, 298 Xinhua Road, Chongchuan District, Nantong, 226002 Jiangsu China

**Keywords:** Acute lung injury, Wogonin, Inflammation, SIRT1, HMGB1 deacetylation

## Abstract

**Background:**

Wogonin (5, 7-dihydroxy-8-methoxyflavone) is a natural di-hydroxyl flavonoid extracted from the root of *Scutellaria baicalensis* Georgi. This paper was intended to investigate the mechanism of action of wogonin in alleviating the inflammation and apoptosis in acute lung injury (ALI).

**Materials and methods:**

Lipopolysaccharide (LPS) was used to establish the in vitro model of ALI. After wogonin treatment, the cell viability and apoptosis of LPS-induced A549 cells were, respectively, measured by CCK-8, TUNEL assays and acridine orange/ethidium bromide dual staining, while the contents of inflammatory cytokines and oxidative stress markers were estimated by RT-qPCR, ELISA assay, western blot analysis and commercial kits. Western blot was also conducted to assess the expression of proteins involved. Subsequently, the effect of wogonin on the sirtuin 1 (SIRT1)-mediated high-mobility group box 1 protein (HMGB1) deacetylation was investigated. SIRT1 inhibitor EX527 was used to evaluate the regulatory effects of wogonin on SIRT1-mediated HMGB1 deacetylation in A549 cells under LPS stimulation.

**Results:**

LPS induced inflammation, oxidative stress and apoptosis of A549 cells, which was abolished by wogonin. It was also found that wogonin promoted the HMGB1 deacetylation, accompanied by upregulated SIRT1 expression. However, SIRT1 inhibitor EX527 partially reversed the protective effects of wogonin on the inflammation and apoptosis of LPS-induced A549 cells.

**Conclusion:**

Wogonin alleviated the inflammation and apoptosis in LPS-induced A549 cells by SIRT1-mediated HMGB1 deacetylation, which might represent the identification of a novel mechanism by which wogonin exerts protective effects on ALI and provide ideas for the application of wogonin to ALI treatment.

## Background

Acute lung injury (ALI) is clinically documented as the result of the pathological states including sepsis, pneumonia, trauma, and acute pancreatitis [1]. It is often seen in patients who are admitted into the intensive care units and shows a high mortality rate [2]. Those who survive from ALI are usually confronted with inferior quality of life [3]. Although achievements have been made in understanding the pathophysiology of ALI, the treatment efficacy of current therapies remains limited, which cannot fulfill the expectations of the patients and their families [4]. Thus, it is of great urgency to identify new therapies or targets for the treatment of ALI.

**Fig. 1:**
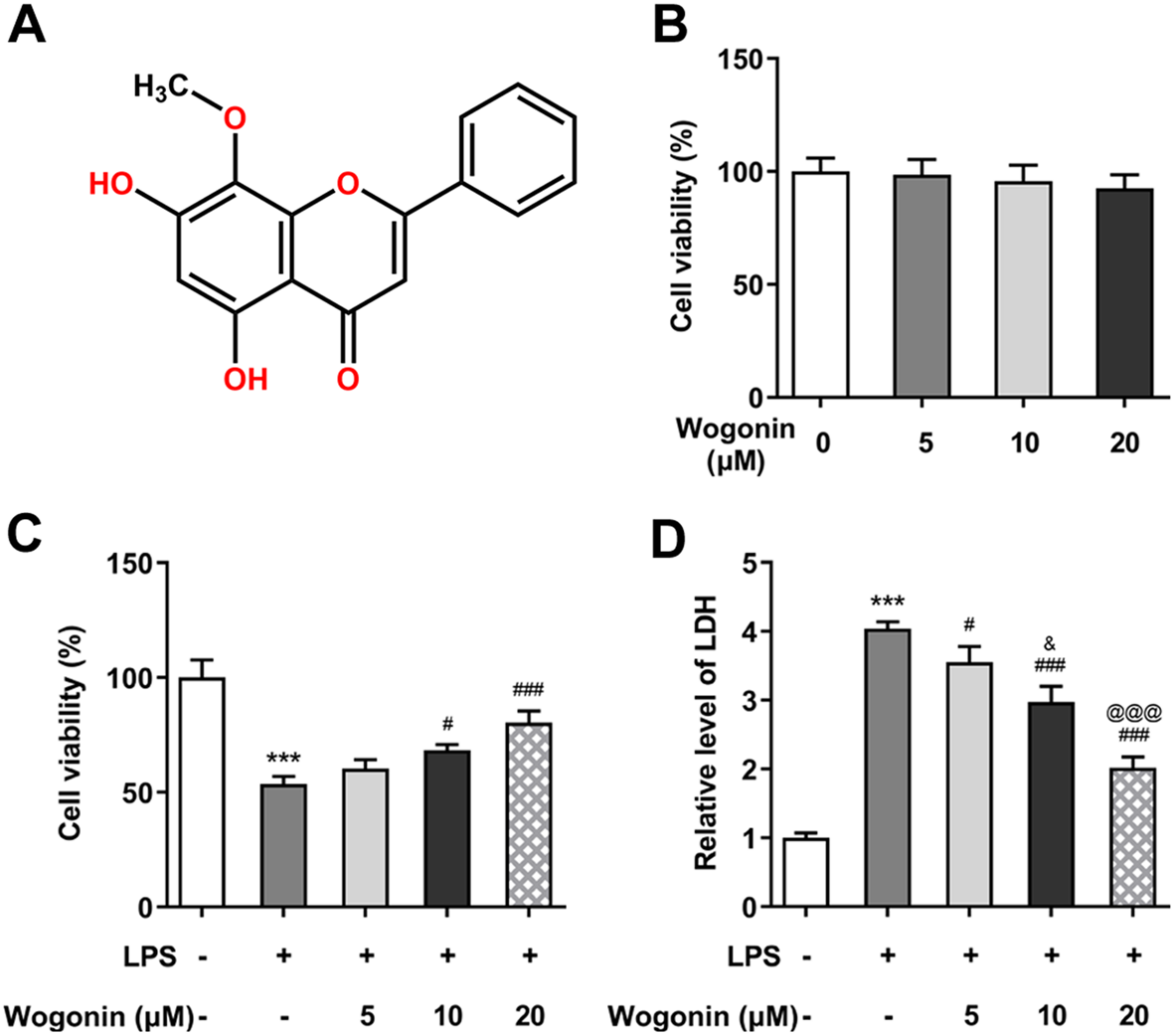
Effect of wogonin on the viability of LPS-induced A549 cells. **A** The chemical structure of wogonin. **B** The viability of A549 cells exposed to wogonin was determined by a CCK-8 assay. **C** The viability of LPS-induced A549 cells exposed to wogonin was estimated by a CCK-8 assay. **D** The LDH activity in LPS-induced A549 cells exposed to wogonin was determined by a LDH assay kit. Data were obtained from three independent experiments (*n* = 3). ***P < 0.001 vs. control; ^#^*P* < 0.05, ^###^*P* < 0.001 vs. LPS; ^&^*P* < 0.05 vs. LPS + 5 μM wogonin; ^@@@^*P* < 0.001 vs. LPS + 10 μM wogonin

Wogonin (5,7-dihydroxy-8-methoxyflavone), the structure of which is shown in Fig. 1A, is a natural di-hydroxyl flavonoid extracted from the root of *Scutellaria baicalensis* Georgi [5]. Its significant anti-inflammatory properties and its application in the treatment of inflammatory diseases have been widely reported by a large number of studies. For instance, wogonin potentially improved the lung edema in the murine and protected them from lipopolysaccharide (LPS)-induced ALI by blocking p38 mitogen activated protein kinase (MAPK) and c-Jun NH(2)-terminal kinase (JNK) phosphorylation [6]. Wogonin also reduced LPS-induced neutrophil infiltration, production of proinflammatory cytokines, expression of adhesion molecules and suppressed LPS-induced ALI in mice [7]. Current evidence suggested that the inflammatory response and ALI induced by LPS could be attenuated as a result of wogonin treatment by peroxisome proliferator-activated receptor gamma (PPARγ)-mediated NF-κB pathway [8]. Emerging study showed that wogonin reduced cecal ligation and puncture (CLP)-induced high-mobility group box 1 protein (HMGB1) production and HMGB1-dependent inflammation, and lowered sepsis-related morbidity and the risk of lung injury [9]. In the LPS-induced in vitro and in vivo ALI model, another report demonstrated that the inhibition of HMGB1 and other inflammatory mediators via ulinastatin evidently alleviated the manifestations of ALI [10]. And previous studies confirmed that LPS-induced human lung epithelial cells (A549) has been widely used as an in vitro model for ALI [11–13].

Sirtuin 1 (SIRT1), the downstream effector of adenosine monophosphate activated protein kinase (AMPK), has been suggested to play significant roles in the regulation of adiponectin release [14]. Wogonin supplementation remarkably increased the AMPK phosphorylation and SIRT1 expression, whereas inhibition of AMPK or SIRT1 reduced the effects of wogonin on the production or release of adiponectin [14]. It was previously reported that SIRT1-modulated HMGB1 deacetylation inhibited acute kidney injury incurred by sepsis [15].

In the present study, we aimed to investigate the action mechanism of wogonin in alleviating the inflammation and apoptosis of LPS-induced human lung epithelial cells, which might provide a more comprehensive insight for the treatment of ALI by wogonin.

## Results

### Effects of wogonin on the viability and apoptosis of LPS-induced A549 cells

**Fig. 2:**
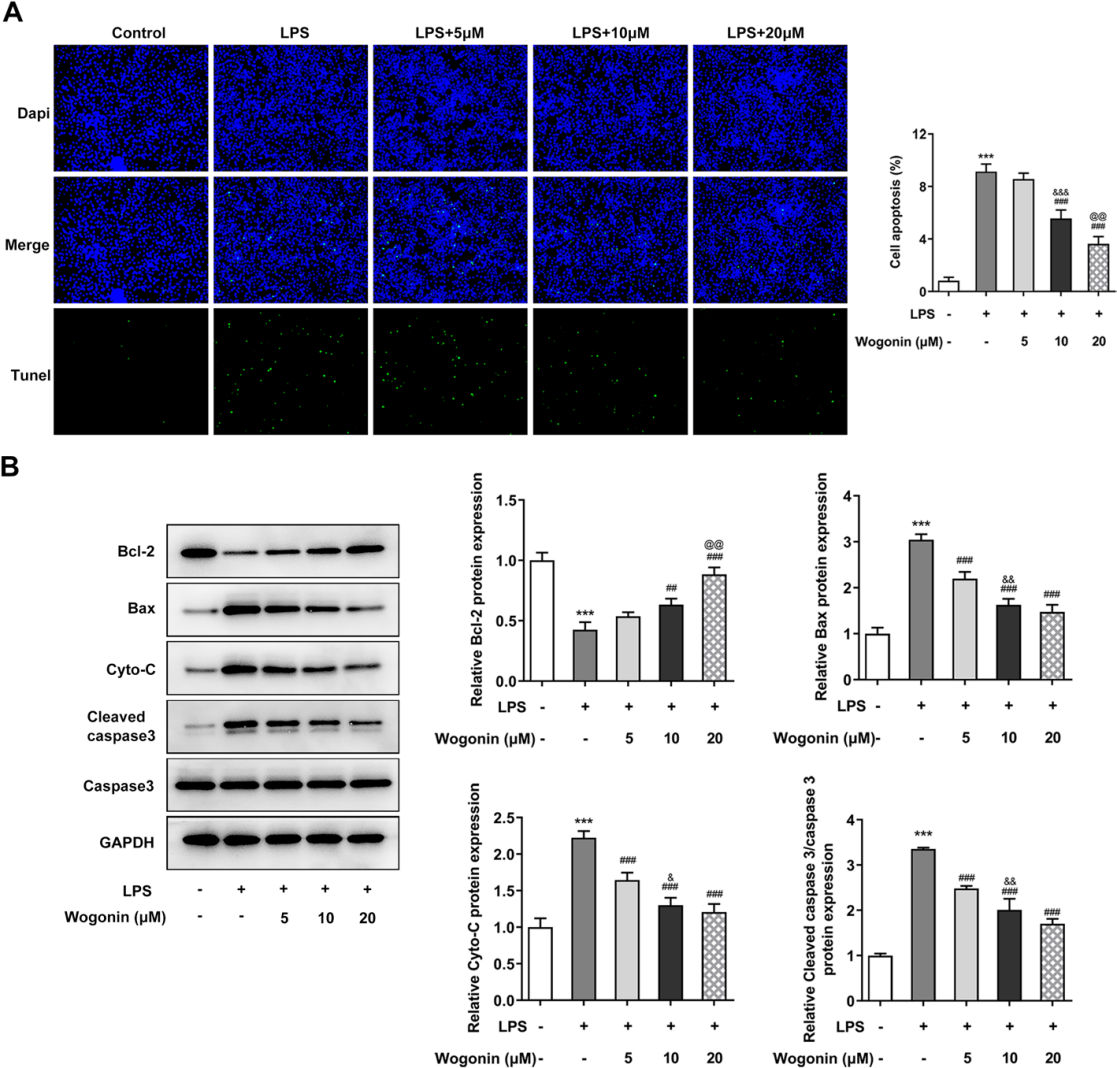
Effect of wogonin on the apoptosis of LPS-induced A549 cells. **A** A TUNEL assay was performed to determine apoptosis of LPS-treated A549 cells upon wogonin exposure. **B** The expression of apoptosis-related proteins in LPS-treated A549 cells upon wogonin exposure was measured by western blot analysis. Magnification, × 200. All experiments were performed in triplicates (*n* = 3). ***P < 0.001 vs. control; ^##^*P* < 0.01, ^###^*P* < 0.001 vs. LPS; ^&^*P* < 0.05, ^&&^*P* < 0.01, ^&&&^*P* < 0.001 vs. LPS + 5 μM wogonin; ^@@^*P* < 0.01 vs. LPS + 10 μM wogonin

**Fig. 3:**
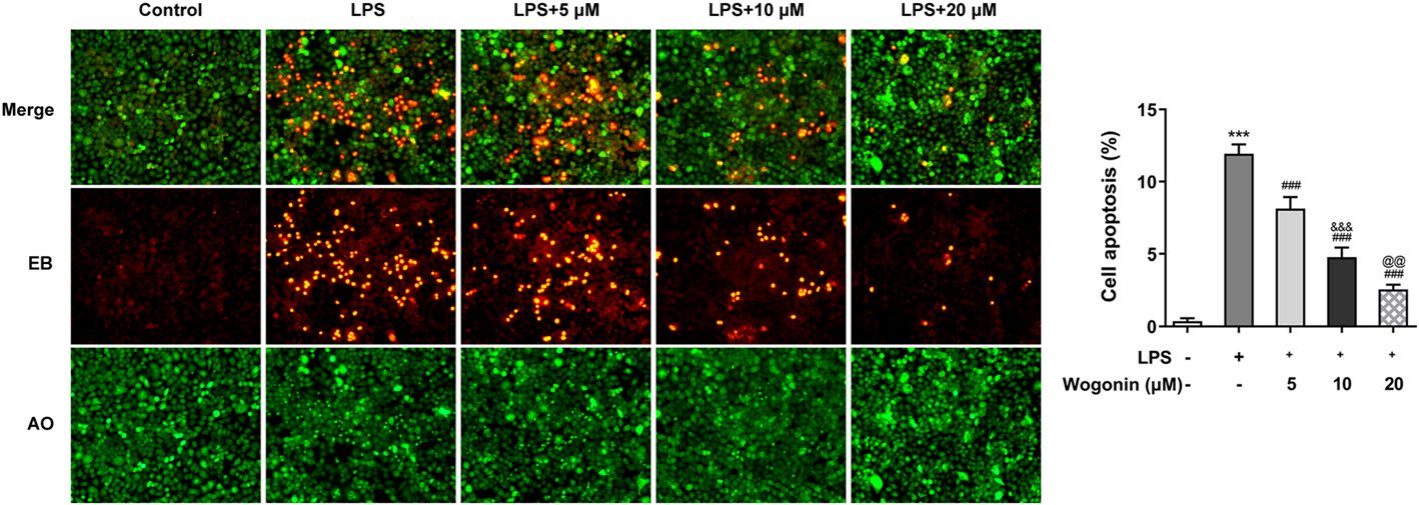
Effect of wogonin on the apoptosis of LPS-induced A549 cells. Cell apoptosis was assessed by acridine orange/ethidium bromide (AO/EB) double fluorescence assays. Results were generated from three independent experiments (*n* = 3). ***P < 0.001 vs. control; ^###^*P* < 0.001 vs. LPS; ^&&&^*P* < 0.001 vs. LPS + 5 μM wogonin; ^@@^*P* < 0.01 vs. LPS + 10 μM wogonin

To observe the effect of wogonin on ALI, we first speculated if the viability of normal A549 cells could be damaged by wogonin. As exhibited in Fig. 1B, wogonin did no harm to A549 cells without receiving any treatment, suggesting the safety of wogonin treatment in A549 cells at the concentrations of 0, 5, 10, and 20 µM. After LPS stimulation for 24 h, the viability of A549 cells was markedly damaged, which was counteracted by wogonin in a concentration-dependent manner (Fig. 1C). Furthermore, results from LDH assay showed that significant increase in LDH activity in LPS-treated A549 cells was reduced after increasing doses of wogonin were administrated (Fig. 1D). Next, we tested the effect of wogonin on the apoptosis of LPS-induced A549 cells. As exhibited in Fig. 2A, TUNEL staining demonstrated that LPS exposure dramatically enhanced the apoptosis of A549 cells compared with the control group, which was reduced by wogonin in a dose-dependent manner. Besides, apparent downregulation in Bcl-2 expression and upregulation in Bax, Cyto-C and cleaved caspase 3 expression were observed in the LPS-exposed group when compared to the control group, which was restored by wogonin treatment (Fig. 2B). Consistently, results of AO/EB double fluorescence assays presented in Fig. 3 suggested that LPS led to the elevated cell apoptosis rate as comparison to the control group, while the EB-positive cells were notably reduced after wogonin addition when compared to the LPS group. Thus, wogonin restores the viability and reduces the apoptosis of A549 cells induced by LPS.

### Effects of wogonin on the inflammation and oxidative stress of LPS-induced A549 cells

**Fig. 4:**
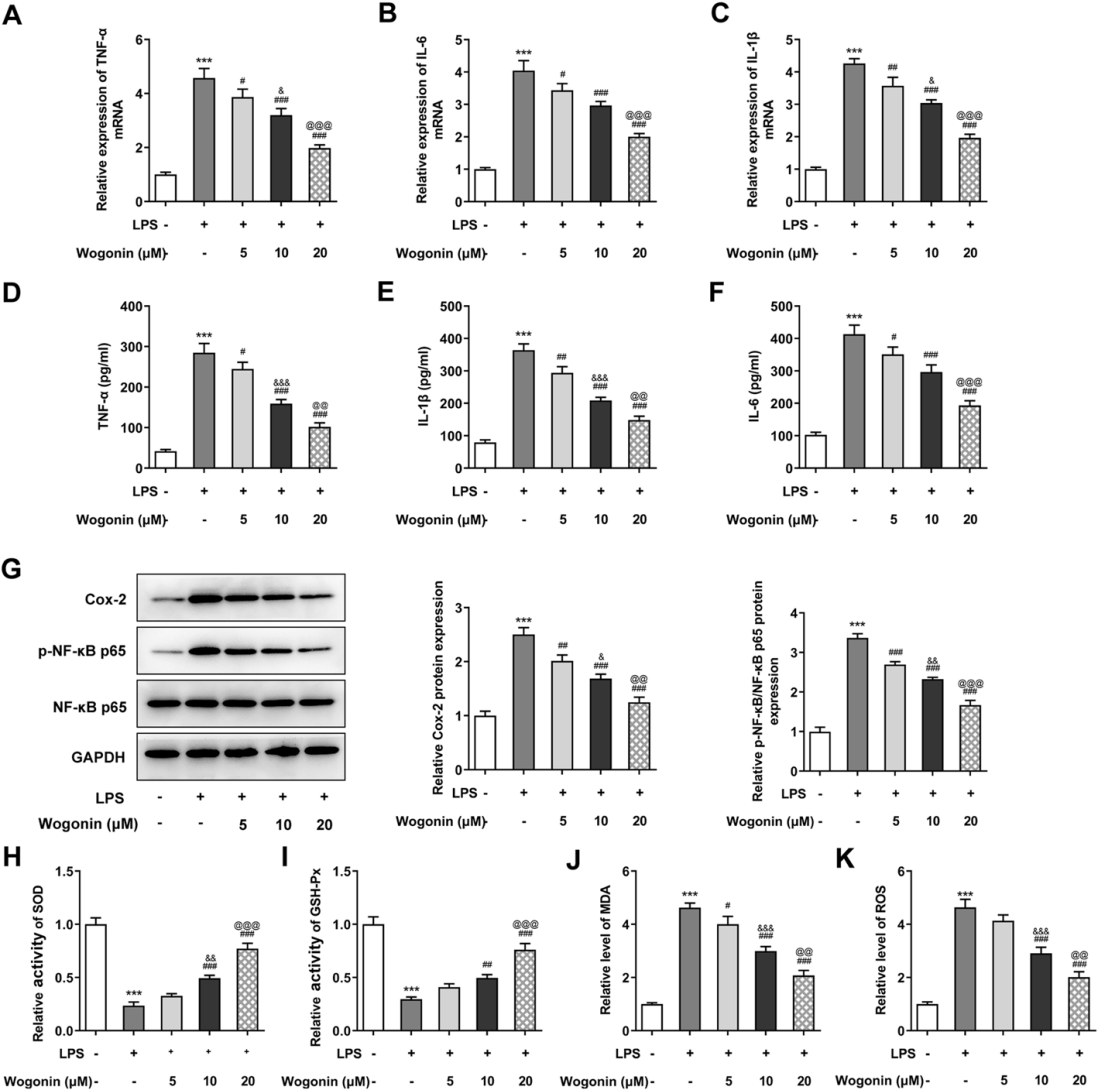
Effect of wogonin on the inflammation and oxidative stress of LPS-induced A549 cells. The mRNA expression levels of **A** TNF-α, **B** IL-6 and **C** IL-1β in LPS-induced A549 cells treated with wogonin were assessed by RT-qPCR. ELISA kits were used to evaluate the concentrations of **D** TNF-α, **E** IL-1β and **F** IL-6 in LPS-induced A549 cells treated with wogonin. **G** The protein expression levels of inflammation-related markers were examined with western blot analysis in LPS-induced A549 cells treated with wogonin. The activities of **H** SOD, **I** GSH-Px and the levels of **J** MDA, **K** ROS were evaluated by kits. Results were generated from three independent experiments (*n* = 3). ***P < 0.001 vs. control; ^#^*P* < 0.05, ^##^*P* < 0.01, ^###^*P* < 0.001 vs. LPS; ^&^*P* < 0.05, ^&&^*P* < 0.01, ^&&&^*P* < 0.001 vs. LPS + 5 μM wogonin; ^@@^*P* < 0.01, ^@@@^*P* < 0.001 vs. LPS + 10 μM wogonin

To explore the anti-inflammatory and anti-oxidant abilities of wogonin in ALI, the inflammation and oxidative stress of LPS-induced A549 cells treated with wogonin were, respectively, detected. As seen in Fig. 4A–C, the mRNA levels of inflammatory cytokines (TNF-α, IL-6 and IL-1β) measured by RT-qPCR rose to notably high levels upon LPS stimulation, whereas wogonin treatment led to the reduction in their expression. Consistently, the results of ELISA indicated that LPS induction led to the significant increase in TNF-α, IL-6 and IL-1β contents compared with the untreated group, which were dose-dependently decreased after wogonin treatment (Fig. 4D–F). Importantly, cyclooxygenase-2 (Cox-2) and phospho-nuclear factor (NF)-κB p65 (p-NF-κB p65), which are inflammation-related markers, were down-regulated by LPS while upregulated by wogonin (Fig. 4G). In addition, LPS resulted in decreased activities of SOD and GSH-Px and increased contents of MDA and ROS, which was reversed by wogonin (Fig. 4H–K). Taken together, wogonin alleviates the inflammation and oxidative stress of LPS-induced A549 cells.

### Regulation of wogonin in SIRT1-mediated HMGB1 deacetylation

**Fig. 5:**
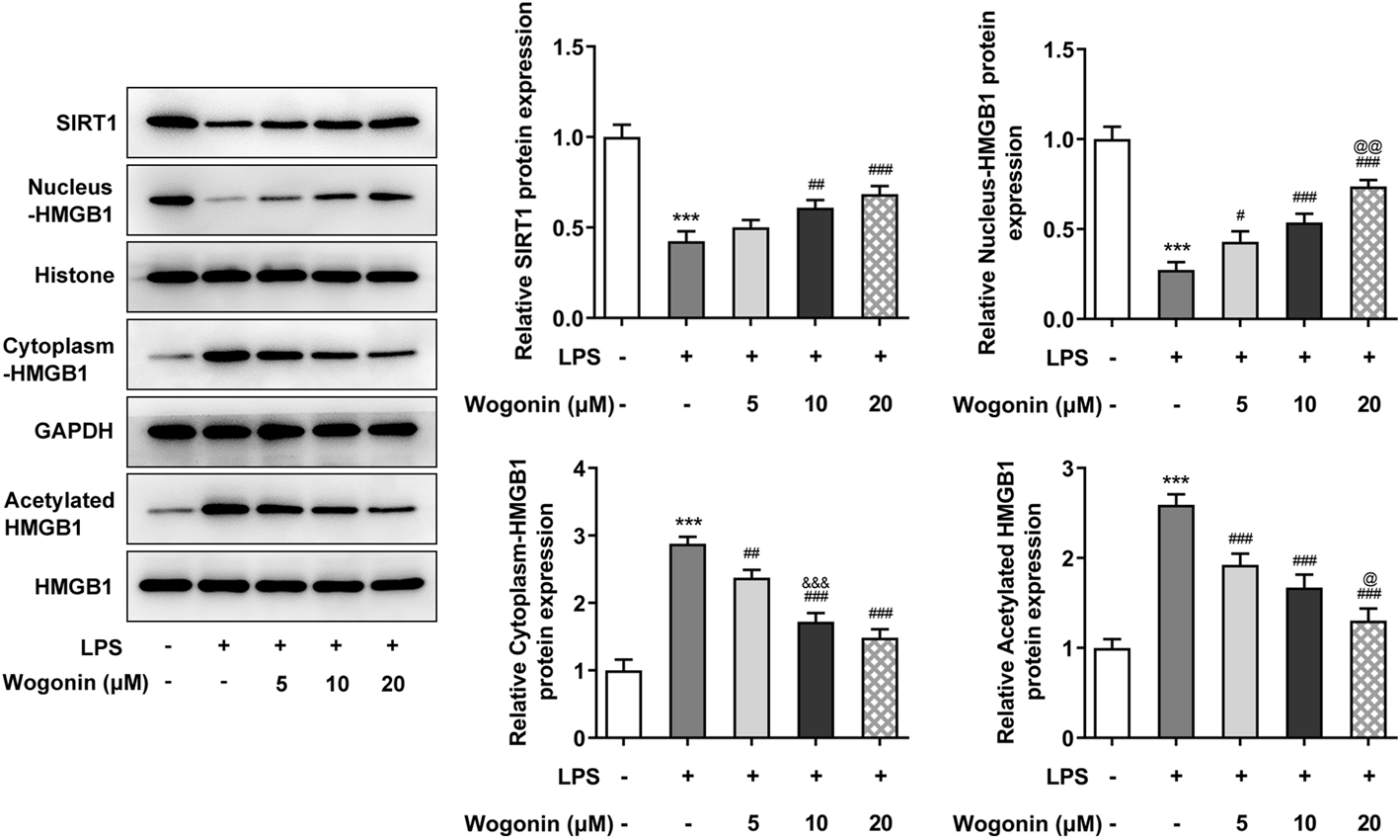
Regulation of wogonin on SIRT1-mediated HMGB1 deacetylation in LPS-induced A549 cells following treatment with wogonin. Western blot assay analyzed the expression of proteins involved in SIRT1-mediated HMGB1 deacetylation. All experiments were performed in triplicates (*n* = 3). ***P < 0.001 vs. control; ^#^*P* < 0.05, ^##^*P* < 0.01, ^###^*P* < 0.001 vs. LPS; ^&&&^*P* < 0.001 vs. LPS + 5 μM wogonin; ^@^*P* < 0.05, ^@@^*P* < 0.01 vs. LPS + 10 μM wogonin

To confirm our speculation that wogonin exerted protective effects on LPS-induced A549 cell injury by SIRT1-mediated HMGB1 deacetylation, we carried out western blot to detect the protein levels of involved factors. Evidently, LPS suppressed the expression of SIRT1 and promoted the translocation of HMGB1 from the nucleus to the cytoplasm, along with HMGB1 acetylation. However, this trend was reversed by wogonin exposure to LPS-induced A549 cells (Fig. 5). Thus, wogonin treatment regulates SIRT1-mediated HMGB1 deacetylation in LPS-induced A549 cells.

### The regulation of SIRT1 inhibitor EX527 on the cellular behaviors of LPS-induced A549 cells treated with wogonin

**Fig. 6:**
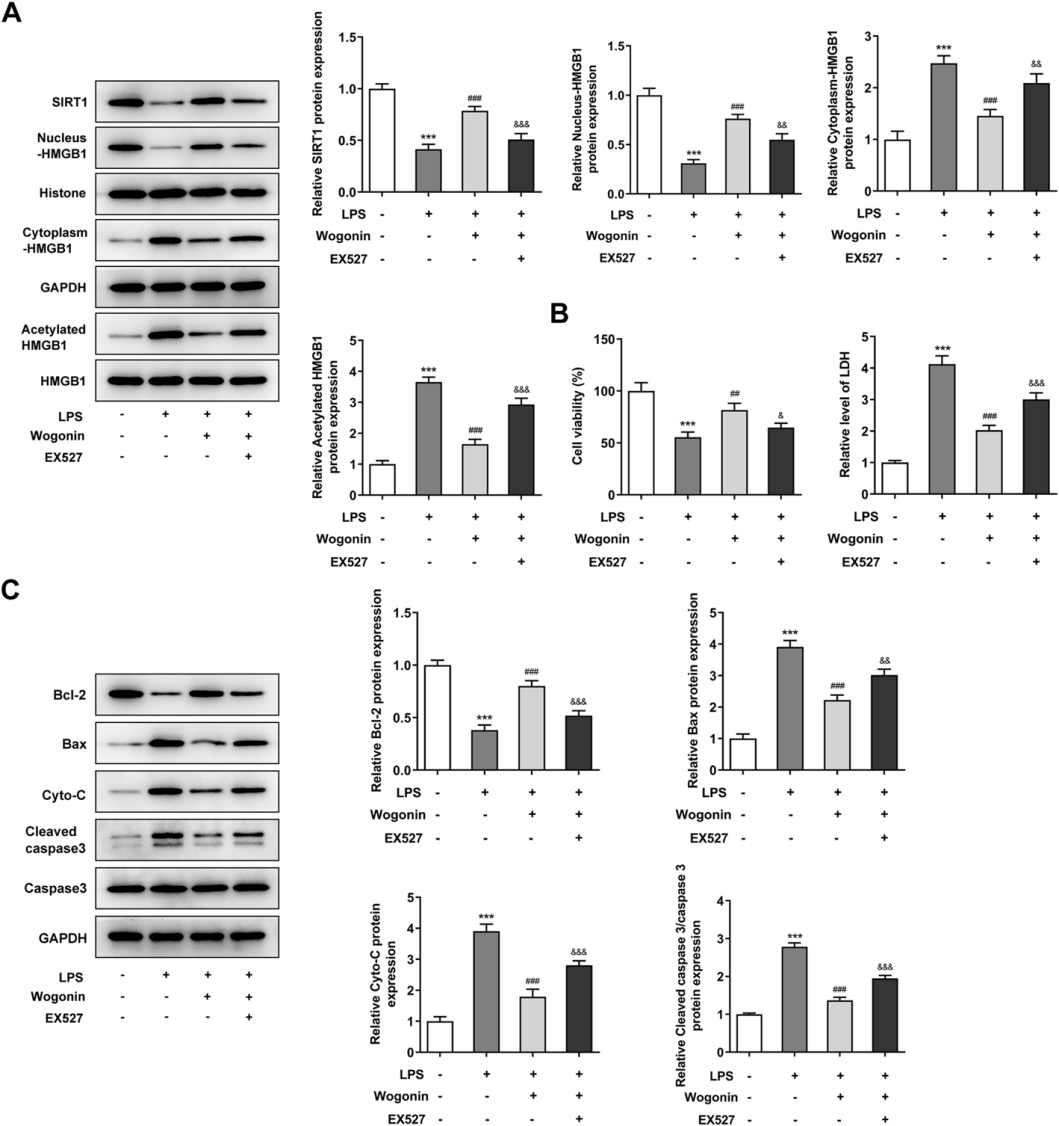
The regulation of SIRT1 inhibitor EX527 on the apoptosis of LPS-induced A549 cells treated with wogonin. **A** Western blot analyzed the expression of proteins involved in SIRT1-mediated HMGB1 deacetylation in LPS-induced A549 cells treated with wogonin and EX527. **B** The viability LPS-induced A549 cells treated with wogonin and EX527 was detected by CCK-8 and LDH assays. **C** The expression of apoptosis-related proteins in LPS-induced A549 cells treated with wogonin and EX527 was tested by western blot analysis. Results were generated from three independent experiments (*n* = 3). ***P < 0.001 vs. control; ^##^*P* < 0.01, ^###^*P* < 0.001 vs. LPS; ^&^*P* < 0.05, ^&&^*P* < 0.01, ^&&&^*P* < 0.001 vs. LPS + wogonin

**Fig. 7:**
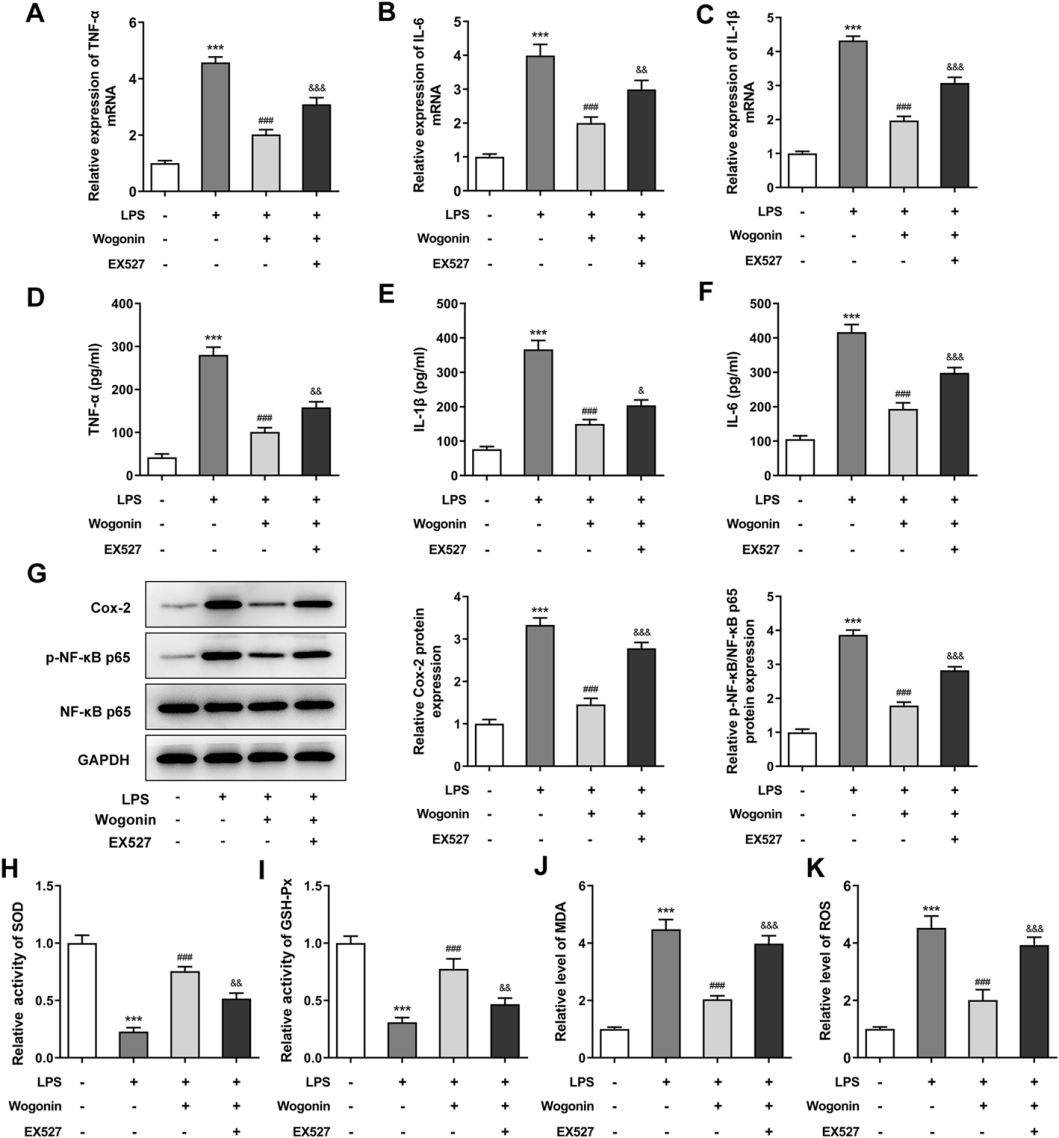
The regulation of SIRT1 inhibitor EX527 on the inflammation and oxidative stress of LPS-induced A549 cells treated with wogonin. The mRNA expression levels of **A** TNF-α, **B** IL-6 and **C** IL-1β were detected by RT-qPCR in LPS-induced A549 cells treated with wogonin and EX527. ELISA kits were used to evaluate the concentrations of **D** TNF-α, **E** IL-1β and **F** IL-6 in LPS-induced A549 cells treated with wogonin and EX527. **G** The expression of inflammation-related proteins was examined with western blot analysis in LPS-induced A549 cells treated with wogonin and EX527. The activities of **H** SOD, **I** GSH-Px and the levels of **J** MDA, **K** ROS were evaluated by kits in LPS-induced A549 cells treated with wogonin and EX527. Data were obtained from three independent experiments (*n* = 3). ***P < 0.001 vs. control; ^###^*P* < 0.001 vs. LPS; ^&^*P* < 0.05, ^&&^*P* < 0.01, ^&&&^*P* < 0.001 vs. LPS + wogonin

To verify the role of SIRT1 in the underlying mechanism by which wogonin impacted the inflammation and apoptosis of LPS-induced A549 cells, we used EX527, an SIRT1 inhibitor, to treat LPS-induced A549 cells for 24 h. As shown in Fig. 6A, the addition of EX527 lessened SIRT1 expression, impeded the nucleo-cytoplasmic transport of HMGB1, and stimulated HMGB1 deacetylation, as compared to LPS + Wogonin group. Further CCK-8 and LDH assays displayed that EX527 damaged the restorative effect of wogonin on the viability of LPS-induced A549 cells (Fig. 6B). Besides, the effects of wogonin on the expression of Bcl-2, Bax, Cyto-C and cleaved caspase 3 expression were partly alleviated by EX527 in LPS-induced A549 cells (Fig. 6C). Finally, the variation trends of the inflammatory factors (TNF-α, IL-6 and IL-1β), inflammation-related proteins (Cox-2 and p-NF-κB p65) and oxidative stress markers (SOD, GSH-Px and MDA) in LPS-induced A549 cells co-treated with wogonin and EX527 demonstrated that EX527 damaged the inhibitory effects of wogonin on the inflammation and oxidative stress in LPS-induced A549 cells (Fig. 7A–K). Therefore, SIRT1 inhibitor EX527 reverses the protective effects of wogonin on the malignant phenotypes of LPS-induced A549 cells.

## Discussion

Currently, many experts have recognized wogonin as potent agents for the treatment of inflammatory diseases. Its efficacy in alleviating inflammatory responses was reportedly attributed to the suppression of NF-κB and activation of nuclear factor erythroid 2-related factor 2 (Nrf2) signaling pathways [16]. Interestingly, previous reports associated the anti-inflammatory effect with the mediation of iNOS and Cox-2 expression or the activation of reactive oxygen species (ROS)/ERK/Nrf2 signaling pathways [5, 17]. Consistent with previous findings displaying no effect of wogonin on chondrocyte viability, we found virtually no significant cytotoxic effect of wogonin on normal A549 cells, supporting our further exploration into the underlying mechanism by which wogonin impacted in vitro ALI cell model [17]. Oxidative stress and inflammation are two major hallmarks in the pathogenesis of ALI, and thus we detected the changes in the oxidative stress indicators and inflammatory cytokines upon wogonin treatment [18]. Intriguingly, inflammation and oxidative stress were both alleviated by wogonin exposure in LPS-treated A549 cell model, as evidenced by the blockade of proinflammatory factor release and the enhanced production of SOD and GSH-Px, as well as decreased production of MDA after wogonin treatment.

HMGB1 emerges as a highly conserved protein that was first identified to modulate sepsis in a murine model [19]. Targeting HMGB1 was considered as a potential therapeutic option for the treatment of sepsis owing to the fact that patients who manifested as prolonged inflammatory responses frequently displayed continued high HMGB1 levels [10]. Recent reports and reviews have paid much attention to the therapeutic effects of HMGB1 on the inflammatory diseases, not limited to sepsis. For instance, it was previously demonstrated that the inhibition of HMGB1 by ulinastatin ameliorated the LPS-induced ALI injury in mice [10, 20]. Following being released from activated monocytes/macrophages, HMGB1 served as a proinflammatory factor via various stimulants [20]. Inflammatory signals like LPS could prompt the acetylation-related translocation of HMGB1 from the nucleus to the cytoplasm [21]. Here, the finding that the translocation of HMGB1 from the nucleus to the cytoplasm upon LPS stimulation was counteracted by wogonin treatment indirectly elucidated that the inflammation of A549 cells triggered by LPS was impeded potently by wogonin.

SIRT1 has been suggested to play a significant role in various biological processes [22]. Depending on its deacetylation targets, alterations in the activity of SIRT1 was expectedly concerned with oxidative stress and inflammation [23]. HMGB1 was recently recognized as a deacetylation target of SIRT1 [20]. Substantial evidence indicated the SIRT1-modulated deacetylation of HMGB1 alleviated inflammation, restored renal function, and prolonged the survival time of mice with sepsis-related acute kidney injury [15]. In an experimental traumatic brain injury study, Omega-3 polyunsaturated fatty acid alleviated the inflammation by modulating microglia polarization through SIRT1-modulated deacetylation of the HMGB1/NF-κB pathway [24]. Further, oleanolic acid prevented rat model from subarachnoid hemorrhage by SIRT1-modulated HMGB1 deacetylation [25]. Inhibition of NLRP3/NF-κB by Aloin via activation of SIRT1 attenuated the LPS-challenged ALI in mice [26]. By regulating SIRT1/HMGB1/NF-κB signaling, kaempferol improved lung injury induced by ischemia–reperfusion via anti-inflammation and anti-oxidative stress [27]. As an important molecule in the downstream of HMGB1, NF-κB is a major signaling pathway involved in the regulation of inflammatory mediators by activating proinflammatory factor genes and releasing a large number of inflammatory factors, including TNF-α, IL-6 and TNF-a [28, 29]. In addition, NF-κB can also participate in the oxidative stress process via regulating the production of ROS and affecting the levels of SOD, MDA and GSH-Px [30]. In the present study, the decreased p-NF-κB p65 expression was observed after wogonin addition, which was restored by SIRT1 inhibitor, EX527. Additionally, we found that deacetylated HMGB1 by wogonin was accompanied by activated SIRT1 expression, which led to our speculation that wogonin might exert protective effects on LPS-induced inflammation of A549 cells by SIRT1-mediated HMGB1 deacetylation. The use of EX527 in the following experiments further demonstrated that inhibition of SIRT1 reversed the protective effects of wogonin on the inflammation of LPS-induced A549 cells.

## Conclusion

In conclusion, we demonstrated that wogonin alleviated the inflammation, oxidative stress and apoptosis of the LPS-induced ALI cell model. This mechanism of action appeared to be owing to the regulation of SIRT1-mediated HMGB1 deacetylation triggered by wogonin. These findings might identify a novel mechanism by which wogonin exerts protective effects on ALI and provide an experimental basis for the application of wogonin to the clinical treatment of ALI.

## Materials and methods

### Cell culture and drugs

Human lung epithelial cells (A549) were purchased from the Cell Bank of Shanghai Institute of Biochemistry and Cell Biology at the Chinese Academy of Sciences (Shanghai, China) and cultured in Dulbecco’s Modified Eagle Medium (DMEM; Gibco, Paisley, UK) containing 10% heat-inactivated fetal bovine serum (Gibco, Paisley, UK), 100 U/ml penicillin, and 100 g/ml streptomycin at 37 °C with 5% CO_2_. The cells were collected after 10 μg/mL LPS (Sigma Chemical Co., St Louis, MO) stimulation for 24 h. Wogonin (purity > 99%), isolated from *Scutellaria baicalensis* Georgi, was bought from Biotic Chemical (Taipei, Taiwan). It was dissolved in dimethylsulfoxide (DMSO), stored at − 20 °C and diluted by DMEM. SIRT1 inhibitor EX527 (10 µM; MedChemExpress, Shanghai, China) was used to treat cells for 24 h before administration according to the previous study [31].

### Cell Counting Kit-8 (CCK-8) assay

After the stimulation of LPS for 24 h and various concentrations of wogonin exposure for another 4 h, cells were seeded into a 96-well plate and cell viability assay was performed by adding 10 µL of CCK-8 reagent (Dojindo, Kumamoto, Japan) into each well at 37 °C for 4 h. The absorbance at 450 nm was monitored using a microplate reader (Bio-Rad Laboratories, Inc.).

### Lactate dehydrogenase (LDH) release assay

The cell supernatant treated with wogonin was seeded in 96-well culture plates and incubated for 24 h at 37 °C with 5% CO_2_ before 100 μL of the Cytotoxicity Detection Kit LDH solution (Roche Diagnostics, France) was added to each well. 15 min later, cell supernatant was diluted, with optical density value monitored at 450 nm.

### Terminal-deoxynucleotidyl transferase-mediated nick end labeling (TUNEL) staining

TUNEL assay was carried out to assess the apoptosis of LPS-induced A549 cells with or without wogonin treatment by Colorimetric TUNEL Apoptosis Assay Kit (Beyotime, Shanghai, China) based on the operation guidelines. Following phosphate buffer saline (PBS) washing for twice and fixation by 4% paraformaldehyde for 0.5 h, 0.3% hydrogen peroxide in PBS was used to incubate cells for another 20 min at room temperature. Cells were then treated by diaminobenzene (DAB) for 10 min and counterstained by hematoxylin (Solarbio, Beijing, China) for 30 s. The apoptotic-positive cells were observed by a fluorescence microscope (Olympus Corporation) and the apoptotic rate was qualified by Image-J software (NIH, Bethesda, MD, USA).

### Acridine orange/ethidium bromide dual staining

Acridine orange/ethidium bromide (AO/EB) double fluorescence assays were performed to evaluate cell apoptosis alteration. In brief, A549 cells were grown on glass coverslips in 24-well plates. The cells were pre-incubated for 24 h with serum-free medium, and then incubated with LPS and various concentrations of wogonin at 37 °C. The cells were washed with PBS and supplied with 5 µL AO/EB mixed solution (100 μg/ml of AO and 100 μg/ml of EB mixed in PBS, Aladdin, China) within 3 min. The cells were observed and taken a photograph at 510 nm excitation wavelength under a fluorescence microscope (Olympus Corporation).

### Western blot analysis

Proteins were extracted from A549 cells using RIPA lysis buffer (Beyotime, Shanghai, China) and the concentrations of proteins were measured by a bicinchoninic acid (BCA) Protein Assay Kit (Thermo Fisher Scientific, USA). Following that, 10% sodium dodecyl sulfate-polyacrylamide gel electrophoresis (SDS-PAGE) electrophoresis was performed to separate 40 µg proteins and the proteins were then transferred to polyvinylidene difluoride (PVDF) membranes. These membranes were then blocked by nonfat milk and then incubated at 4 °C overnight with primary antibodies. Afterwards, HRP-conjugated secondary antibody was used to incubate the membranes for 1.5 h at room temperature. Finally, bands were visualized by an enhanced chemiluminescence (ECL) kit (Amersham Biosciences, Buckinghamshire, UK), while band intensity was monitored using Image-J software (NIH, Bethesda, MD, USA). GAPDH was considered as an internal reference.

### Reverse transcription-quantitative PCR (RT-qPCR)

A549 cells were lysed in 1 ml TRIzol® reagent (Invitrogen, Carlsbad, CA, USA) for the extraction of total RNA. After the collection of total RNA, reverse transcription was conducted for complementary DNA (cDNA) synthesis by PrimeScript RT reagent kit (Takara Bio, Inc.). The conditions were as follows: 50 °C for 15 min, 85 °C for 5 s and preservation at 4 °C. SYBR Premix Ex Taq TM (TaKaRa, Japan) was used to set the PCR reaction conditions and reaction system based on the suggestions of the manufacturer. An ABI 7500 instrument (AB-4351107; Applied Biosystems; Thermo Fisher Scientific, Inc.) was used for qPCR. The following thermocycling conditions were used: initial denaturation at 95 °C for 10 min; followed by 40 cycles of denaturation at 95 °C for 15 s and annealing at 60 °C for 1 min; and a final extension of 10 min at 72 °C. GAPDH was accepted as the normalization control of relative gene expression. Calculation of relative gene expression was conducted by 2^−ΔΔCt^ method.

### Enzyme-linked immunosorbent assay (ELISA)

The cellular levels of interleukin-6 (IL-6), IL-1β and tumor necrosis factor-α (TNF-α) in the cell supernatant were measured using IL-6 ELISA Kit, IL-1β ELISA Kit and TNF-α ELISA Kit based on the suggestions of the manufacturer (Shanghai XiTang Biotechnology, Shanghai, China).

### Detection of oxidative stress

For the detection of oxidative stress, the activities of oxidative stress markers including malondialdehyde (MDA), reactive oxygen species (ROS), glutathione peroxidase (GSH-Px) and superoxide dismutase (SOD) were detected following the manufacturer’s instructions (Nanjing Jiancheng Biotechnology Institute, China).

### Statistical analysis

All data were shown as mean ± standard deviation (SD) of at least three independent experiments. For comparisons among multiple groups, one-way analysis of variance (ANOVA) with Tukey’s post hoc test was carried out, while those between two groups were conducted by Student’s *t*-test. *P* < 0.05 was deemed as statistically significant.

## Data Availability

The experimental data will be available on the request.

## Abbreviations

ALI: Acute lung injury
LPS: Lipopolysaccharide
HMGB1: High-mobility group box 1 protein
PPARγ: Peroxisome proliferator-activated receptor gamma
MAPK: Mitogen Activated Protein Kinase
JNK: C-Jun NH(2)-terminal kinase
CLP: Cecal ligation and puncture
DMEM: Dulbecco’s modified Eagle medium
DMSO: Dimethylsulfoxide
CCK-8: Cell counting kit-8
LDH: Lactate dehydrogenase
TUNEL: Terminal-deoxynucleotidyl transferase-mediated nick end labeling
PBS: Phosphate buffer saline
DAB: Diaminobenzene
AO/EB: Acridine orange/ethidium bromide
BCA: Bicinchoninic acid
SDS-PAGE: Sodium dodecyl sulfate-polyacrylamide gel electrophoresis
PVDF: Polyvinylidene difluoride
ECL: Enhanced chemiluminescence
cDNA: Complementary DNA
ELISA: Enzyme-linked immunosorbent assay
IL: Interleukin
TNF-α: Tumor necrosis factor-α
MDA: Malondialdehyde
GSH-Px: Glutathione peroxidase
SOD: Superoxide dismutase
SD: Standard deviation
ANOVA: Analysis of variance
Cox-2: Cyclooxygenase-2
Nrf2: Nuclear factor erythroid 2-related factor 2
ROS: Reactive oxygen species
